# Gastric Bypass Surgeries in New Hampshire, 1996-2007

**DOI:** 10.5888/pcd9.110089

**Published:** 2012-03-15

**Authors:** Sai S. Cherala

**Affiliations:** Office of Health Statistics and Data Management, Bureau of Public Health Statistics and Informatics, Division of Public Health Services, Department of Health and Human Services

## Abstract

**Introduction:**

Obesity is a national epidemic. Gastric bypass surgery may be the only option that provides significant long-term weight loss for people who are morbidly obese (body mass index [BMI] ≥40 kg/m^2^) or for people who have a BMI of 35 or higher and have an obesity-related comorbidity. The objective of this study was to assess trends in gastric bypass surgery in New Hampshire.

**Methods:**

Data from 1996 to 2007 from the New Hampshire Inpatient Hospital Discharge data set were analyzed. Records for patients with a gastric bypass surgery code were identified, and data on patients and hospitalizations were collected. A joinpoint regression model was used to analyze trends in surgery rates. Differences between patients and payer types were analyzed by using the Cochran–Mantel–Haenszel χ^2^ test.

**Results:**

The annual rate of gastric bypass surgery increased significantly from 3.3 to 22.4 per 100,000 adults between 1996 and 2007. The in-hospital death rate decreased significantly from 11% in 1996 to 1% in 2007. A greater proportion of women (78.1% during the study period) than men had this surgery. The average charge of a surgery decreased significantly from $44,484 in 1996 to $43,907 in 2007; by 2007, total annual charges were $13.9 million. Since 1996, private or "other" payers have been charged for nearly 80% of the total discharges.

**Conclusion:**

The number of gastric bypass surgeries has increased in New Hampshire, and so have their cost. These increases may reflect a shortage in effective primary care and preventive measures to address the obesity epidemic.

## Introduction

Obesity is a growing public health concern in the United States (1,2). The prevalence of obesity increased significantly between 1970 and 2008 among adults (from 15% to 34%), and children and adolescents (from 5% to 17%) (3-5). The prevalence of obesity in New Hampshire has followed a similar trend: the prevalence of morbidly obese adults (body mass index [BMI] ≥40 kg/m^2^) increased significantly from 1.3% in 1996 to 2.5% in 2007. In 2007, 25% of New Hampshire adults were obese (BMI ≥30) (S. Knight, MSPH, New Hampshire Department of Health and Human Services, e-mail communication, January 2011).

Strategies have emerged to counter the increasing rates of obesity. At the population level, strategies include social marketing campaigns, environmental changes to encourage increased physical activity, and regulations, such as bans on sugar-sweetened beverages (6). At the individual level, strategies range from books and television shows on exercise and diet to surgery. Bariatric surgery has become a popular method of treating obesity; gastric bypass surgery has emerged as the most widely used of these surgical procedures (7-9).

The National Heart, Lung, and Blood Institute's Clinical Guidelines recommend weight-loss surgery as "an option for well-informed and motivated patients who have clinically severe obesity (BMI ≥40) or a BMI ≥35 and serious comorbid conditions" (10).

Weight-loss surgery provides medically significant sustained weight loss for more than 5 years in most patients (11). Postoperative complications vary by type of surgery, baseline weight, and overall health of the patient. In the prospective Swedish Obese Subjects study, overall death rates in the surgery group decreased during an average 10.9 years of follow-up, compared with matched subjects in the control group (12). Most patients fared well after surgery and showed a significant improvement in quality of life.

The objective of this study was to assess trends in gastric bypass surgery in New Hampshire.

## Methods

Inpatient and emergency department acute care hospital discharge data are collected under New Hampshire Revised Statutes Annotated (RSA) 126:25, which requires all facilities in New Hampshire to report discharge data to the New Hampshire Department of Health and Human Services. Records for New Hampshire residents who are discharged in Massachusetts, Maine, and Vermont are obtained by the department through special interstate data exchange agreements with the agencies in those states responsible for collection of discharge data. In 2009, 30 licensed general and specialty acute care hospitals in New Hampshire submitted their inpatient hospital data.

For this study, inpatient discharge data from the New Hampshire Inpatient Hospital Discharge data set were analyzed; the data set captures hospitalization-related information, including information on charges, for all New Hampshire residents hospitalized in New Hampshire, Maine, Massachusetts, and Vermont. The data set provides high-quality data on hospitalizations, procedures, and diagnoses, and data are comparable from year to year. Data from 1996 through 2007 were collected for this study. Age-adjusted rates (per 100,000 area population) of hospitalizations related to gastric bypass surgery, by year, sex, and age, and the proportion of hospitalizations by expected primary payer were calculated. Institutional review board approval was not required for this study because it was done as part of public health surveillance.

### Variables

Diagnoses and procedures for the hospitalizations were coded according to the *International Classification of Diseases, Ninth Revision, Clinical Modification* (ICD-9-CM) (13). Discharge records with a gastric bypass surgery code of 44.31 (gastroenterostomy without gastrectomy; high gastric bypass), 44.38 (laparoscopic gastroenterostomy), 44.39 (gastroenterostomy without gastrectomy, other gastroenterostomy), or 44.95 (laparoscopic gastric restrictive procedure) in any procedure field were identified. Data on patients (ie, year of hospitalization, sex, and age) and hospitalizations (ie, length of stay, mean hospital charges, expected primary payer, and in-hospital death rate) were collected. The patient's insurer at the time of data collection was identified as the expected payer. Payers were identified as Medicare, Medicaid, private or "other" payers, and self-pay.

Data were reviewed to determine the relative proportions of the 2 main indications for surgery: a principal diagnosis of morbid obesity (ICD-9-CM, 278.01) or a principal diagnosis of an obesity-related comorbid condition, or both. The following obesity-related comorbid conditions, selected from the Charlson comorbidity index (14), were included in the analysis: cereberovascular disease, congestive heart failure, chronic pulmonary disease, diabetes, hypertension, liver disease (moderate to severe), myocardial infarction, peripheral vascular disease, and moderate to severe renal disease. Other conditions, such as endocrine and mental disorders, were also examined.

Discharge records for people with an unknown residence or for people residing outside New Hampshire were excluded from this analysis. Records that had diagnosis codes from 150.0 to 159.9, corresponding to malignant neoplasm of digestive organs and peritoneum, were also excluded from analysis. Information on race and ethnicity is not consistently collected through the New Hampshire Inpatient Hospital Discharge data set, so analysis by race/ethnicity could not be performed.

### Statistical analysis

The estimated annual number of gastric bypass surgeries was derived by counting the number of discharge records coded with a gastric bypass surgery in any of the procedure fields in the data. Annual population rates were age-adjusted for the 2000 US population. The estimated rates were not based on the population of overweight or obese residents and were not adjusted for the number of adults who may have previously had a gastric bypass surgery.

Secondary analysis included calculating by year the proportion of gastric bypass surgeries associated with each type of payer, the total charges of surgery for each payer, and the frequency of each comorbidity. All charges were adjusted for inflation for 2007 by using the US Bureau of Labor Statistics inflation calculator, which uses the average Consumer Price Index for a given calendar year.

A joinpoint regression model was used to analyze trends in the annual rates of gastric bypass surgery. The differences between female and male discharged patients and differences among payer types were analyzed by using the Cochran–Mantel–Haenszel χ^2^ test (15). Statistical significance was set at a *P* value of less than .05. All statistical tests were 2-sided, and all analyses were performed with SAS for Windows software (SAS Institute, Inc, Cary, North Carolina).

## Results

From 1996 through 2007, the estimated annual number of gastric bypass surgeries increased significantly from 37 surgeries in 1996 to 318 in 2007 (Table 1), and the estimated annual rate of gastric bypass surgeries increased significantly from 3.3 to 22.4 per 100,000 people (Figure). The rate increased significantly by age in the 2 younger age categories but most notably among people aged 25 to 64 years: from 2.7 per 100,000 in 1996 to 39.2 per 100,000 in 2007.

**Figure.**
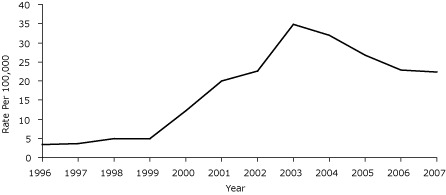
Age-adjusted gastric bypass hospitalization rates in New Hampshire, 1996-2007. Source: New Hampshire Inpatient Discharge Data, New Hampshire Office of Health Statistics and Data Management.

**Table:** 

Year	Age-adjusted Rate Per 100,000
1996	3.3
1997	3.7
1998	4.9
1999	4.8
2000	12.2
2001	19.9
2002	22.6
2003	34.8
2004	32.0
2005	26.7
2006	22.9
2007	22.4

A greater proportion of women (78.1% during the study period) than men had this surgery. The estimated total number of surgeries performed on women (2,202 surgeries) was more than 3 times the number performed on men (618 surgeries). The mean age for surgery was 46 years; nearly 29% of surgeries were performed in patients aged 35 to 44 years.

The mean length of hospital stay decreased from 19 days in 1996 to 5 days in 2007. The in-hospital death rate decreased from 11% in 1996 to 1% in 2007.

The proportion of private or "other" insurers as expected payers increased significantly from 32% in 1996 to 81% in 2007, whereas the proportion of Medicare and Medicaid as expected payers declined (Table 2). The significant increase in the number of gastric bypass surgeries translated into gradually increasing charges — from $1.6 million in 1996 to $13.9 million in 2007. During the study period, the charges for gastric bypass surgeries totaled approximately $99.5 million, and the average charge per discharge decreased significantly from $44,484 in 1996 to $43,907 in 2007. The estimated charge per discharge varied widely, ranging from $24,656 in 2001 to $60,386 in 2002. Since 1996, private or "other" payers have been charged for nearly 80% of the total discharges.

Overall, the most common comorbid conditions associated with gastric bypass surgery were hypertension and endocrine disorders, excluding diabetes (Table 3). Of all gastric bypass discharges, 2,425 (86%) had a principal diagnosis of morbid obesity, and approximately 8% had a principal diagnosis related to a comorbid condition. Approximately 6% of discharges had neither a principal diagnosis of morbid obesity nor a comorbid condition.

## Discussion

The rate of gastric bypass surgery for the treatment of obesity increased significantly in New Hampshire from 1996 through 2007. Previous national population-based studies reported similar findings (16-18). Surgery charges in New Hampshire also increased, reaching $13.9 million in 2007. Although charges increased significantly among all payers, charges shifted from Medicare, which accounted for 51.4% of the discharges in 1996, to private or "other" payers, which accounted for 81.4% of the discharges in 2007. This shift in payers mirrors a national pattern (16-17).

The potential for long-term weight loss may explain the rise in the number of gastric bypass surgeries in New Hampshire. Obesity is a complex condition that has many causes, including genetic and environmental factors that are largely beyond a person's control, and many health, social, psychological, and economic consequences (19). The current US generation may have a shorter life expectancy than the previous one if the rise in obesity continues (20). Ideally, the results of weight-loss methods should be long term (ie >10 y) (21). The amount of weight loss achieved through dieting is often small and difficult to sustain. A meta-analysis showed that patients who underwent gastric bypass surgery lost an average of 61.6% of excess body weight (22). Another study showed that gastric bypass surgery typically resulted in the loss of 50% of excess body weight within the first year, and the effect was often sustained at 10 years (23). Although gastric bypass surgery requires lifestyle changes and poses health risk, including death, the resulting long-term weight loss may be cost-effective and improve health. Most patients are able to reverse diabetes, control hypertension, increase mobility, recover fertility, and improve their overall quality of life (10).

Another explanation for the increase in rates in New Hampshire may be the approval of the laparoscopic-adjustable gastric banding surgical technique by the US Food and Drug Administration in 2001 (24). Although this procedure is more costly and time-consuming than gastric bypass surgery, it is less invasive, and patient recovery times are shorter.

In 2004, the Centers for Medicare and Medicaid Services changed its position on obesity (25); it began to consider obesity as a  treatable illness and to pay for gastric bypass surgeries. This change may also explain the increase in surgery rates after 2004. In 2007, only 11% of adults aged 25 to 64 (the predominant group undergoing gastric bypass surgery) were beneficiaries of either Medicaid or Medicare (26). Among people aged 55 years or older, approximately 47% are obese and 5% are extremely obese (S. Knight, MSPH, New Hampshire Department of Health and Human Services, e-mail communication, January 2011). Some extremely obese people are eligible for bariatric surgery based on BMI criteria alone and will soon be eligible for Medicare benefits based on age criteria if they are not enrolled already (16).

The obesity epidemic in New Hampshire partially explains the observed increase in the frequency of gastric bypass surgeries. Other explanations could be increases in the following: the rates of comorbidities among people with a BMI of 35 or more; the availability of the surgery itself, made possible by more trained physicians and more hospitals with the resources to offer it; willingness among eligible patients to undergo the procedure as it becomes more commonplace and the related rates of illness and death decrease; and insurance coverage in public or private health plans. Private payers in New Hampshire have been bearing an increasingly larger share of the costs of surgery; coverage for gastric bypass surgery among private payers may have increased. Because gastric bypass surgery has shown long-term success, it is becoming a more valuable option for private insurance plans and employers (27).

The increase in gastric bypass surgeries may reflect a shortage in effective primary care and preventive measures to address the obesity epidemic. Some questions raised by this analysis need to be answered in a broader arena of public health and research: How much is invested in attempts at lifestyle change in morbidly obese adults before surgery is performed? What percentage of obese adults are actively counseled by medical providers? What percentage are taking steps to lose weight, and what steps are they taking?

This study has limitations. Procedures or diagnoses may have been misclassified because of a popular perception that insurance companies will not reimburse costs on the basis of a diagnosis of obesity (16). Another potential limitation is that the hospital discharge data represent the number of hospitalizations, not the number of patients. The data did not identify patients who may have undergone multiple surgeries, so a single patient could be represented by more than 1 discharge record. The data set also did not allow identification of the number of claims that were paid, denied, or reversed. The charges reflected by the discharge data set may not include both the physician and facility charges. Finally, current use of gastric bypass surgery may be changing. These limitations may have resulted in undercounting or overcounting and affected the calculation of rates and proportions. Despite these limitations, the findings of this study were similar to those of other national studies (28).

As the obesity epidemic continues, patients and their families, health care providers, and insurance companies may look increasingly to gastric bypass surgery as a practical and cost-effective option for long-term weight loss. The public health community needs to develop more effective primary care and preventive interventions to reach obese people before surgical remedies are necessary.

## Figures and Tables

**Table 1.** Gastric Bypass Surgeries in New Hampshire, 1996-2007^a^

Characteristic	1996	2007	1996-2007	*P* Value^b^
Total surgeries performed, n	37	318	2,820	<.001
Surgery rate per 100,000 population	3.3	22.4	17.9	
Mean age of all patients, y	61.4	47.2	45.9	
Mean age of male patients, y	56	49.7	50.6	
Mean age of female patients, y	64	46.4	44.5	
Surgeries by female sex, %	67.6	75.8	78.1	<.001
Length of hospital stay, mean, d	19	5	5	.009
In-hospital death rate, %	11	1	2	.15
**Age-specific surgery rate, %**				
0-24 y	0.2	0.9	1.4	.006
25-64 y	2.7	39.2	30.3	<.001
≥65 y	13.8	15	13.4	.24

a Source: New Hampshire Inpatient Discharge Data, New Hampshire Office of Health Statistics and Data Management.

b Determined by joinpoint regression for 1996-2007.

**Table 2.** Gastric Bypass Surgery Charges by Expected Primary Payer in New Hampshire, 1996-2007^a^

Characteristic	1996		2007	1996-2007	*P* Value^b^
Total charges, $, millions	1.6		13.9	99.5	.01
Average charge per discharge, $	44,484		43,907	35,300	.01
**Proportion of discharges by payer, %**					
Medicaid	10.8	3.1		4.7	<.001
Medicare	51.4	14.2		14.1	
Private/other	32.4	81.4		79.9	
Self-pay	5.4	1.3		1.4	
**Proportion of charge by payer, %**					
Medicaid	5.2		1.8	4.6	<.001
Medicare	60.4		21.3	19.3	
Private/other	30.2		76.3	75.1	
Self-pay	4.2		0.6	1.0	

a Source: New Hampshire Inpatient Hospital Discharge Data, New Hampshire Office of Health Statistics and Data Management.

b Determined by joinpoint regression for 1996-2007.

**Table 3.** Most Common Comorbidities for People Undergoing Gastric Bypass Surgery (N = 2,820) in New Hampshire 1996-2007^a^

**Condition**	%
Hypertension	30.3
Endocrine disorder, excluding diabetes	26.0
Diabetes	17.2
Musculoskeletal system disorder	16.0
Esophageal reflux	15.0
Asthma and chronic bronchitis	13.2
Mental disorder, including psychosis	12.7
Cholelithiasis (gall stones)	8.5
Malignant neoplasm	8.5
Heart disease	8.1
Chronic liver disease	4.6
Obstructive sleep apnea	3.4

a Source: New Hampshire Inpatient Discharge Data, New Hampshire Office of Health Statistics and Data Management.
